# Feasibility of multifrequency MR elastography of the seminal vesicles in healthy men, benign prostatic hyperplasia, and prostate cancer

**DOI:** 10.1186/s41747-026-00713-2

**Published:** 2026-04-21

**Authors:** Kevin Vatter Davis, Hans-Martin Thiess, Patrick Asbach, Eugenio Tiberi, Timm Denecke, Bernd Hamm, Bernhard Gebauer, Jens Vogel-Claussen, Ingolf Sack, Rolf Reiter

**Affiliations:** 1 Department of Radiology, Charité–Universitätsmedizin Berlin, corporate member of Freie Universität Berlin and Humboldt-Universität zu Berlin, Hindenburgdamm 30, Berlin, Germany; 2 Department of Urology, BG Unfallkrankenhaus Berlin, Berlin, Germany; 3 Department of Diagnostic and Interventional Radiology, University of Leipzig, Leipzig, Germany; 4 Berlin Institute of Health at Charité–Universitätsmedizin Berlin, BIH Biomedical Innovation Academy, BIH Charité Digital Clinician Scientist Program, Charitéplatz 1, Berlin, Germany

**Keywords:** Elasticity imaging techniques, Magnetic resonance imaging, Prostatic hyperplasia, Prostatic neoplasms, Seminal vesicles

## Abstract

**Objective:**

The seminal vesicles (SVs) are important for reproductive health and prostate cancer (PCa) staging. Nevertheless, their biophysical properties remain largely unexplored. This secondary analysis investigates the feasibility and potential of multifrequency magnetic resonance elastography (MRE) in characterizing viscoelastic properties of the SVs across healthy subjects, men with benign prostatic hyperplasia (BPH), and patients with PCa.

**Materials and methods:**

Sixty-two male participants (24 healthy, 25 BPH, 13 PCa) underwent pelvic MRE and diffusion-weighted imaging (DWI). We assessed shear wave speed (SWS), phase angle φ, apparent diffusion coefficient (ADC) and normalized ADC (nADC). Statistical comparisons across groups were performed using Kruskal–Wallis and Dunn tests. Test-retest and bilateral agreement were analyzed using intraclass correlation coefficients (ICCs).

**Results:**

MRE of the SVs was technically feasible with excellent reproducibility, particularly for φ (ICC = 0.989), which was also significantly higher in BPH (*p* = 0.022) and PCa patients (*p* = 0.019), compared to healthy controls, showing elevated tissue fluidity in disease. While SWS and ADC did not differ significantly across groups, nADC was significantly reduced in BPH and PCa patients compared to healthy subjects (*p* < 0.001). No significant differences were detected between PCa patients with and without seminal vesicle invasion.

**Conclusion:**

Multifrequency MRE of the SVs is feasible and highly reproducible, allowing reliable assessment of their viscoelastic properties. Tissue fluidity may serve as a sensitive imaging biomarker. Further studies are warranted to explore the role of SV fluidity in disease progression and diagnosis.

**Relevance statement:**

Multifrequency MRE enables the *in vivo* viscoelastic characterization of the SVs. It demonstrates disease-related increases in tissue fluidity in BPH and PCa. These findings showcase φ as a promising imaging biomarker for pathologies of the SVs and the prostate.

**Key Points:**

- Multifrequency MRE provides an *in vivo* assessment of SVs biomechanics across healthy men and patients with BPH or PCa.
- Across all mechanical parameters, tissue fluidity (φ) demonstrates the highest sensitivity to pathological changes.
- The excellent reproducibility of φ underscores its potential use in future diagnostics targeting prostate and SVs disease.

**Graphical Abstract:**

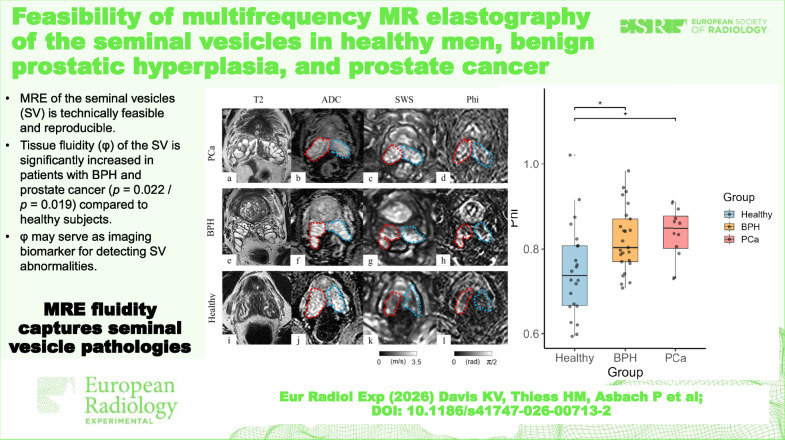

## Background

The seminal vesicles (SVs) are an essential component of the male reproductive system, contributing to semen composition. Their morphology has been associated with libido and even erectile dysfunction. Their secretions exhibit antibacterial properties, modulate immune responses as well as smooth muscle contractions within the female genital tract, and influence sperm motility [1–4]. Anatomically and functionally, the SVs are linked to the prostate [5].

Prostate cancer (PCa) is one of the most prevalent cancers in men and a leading cause of cancer-related mortality worldwide [6–12]. The advent of multiparametric magnetic resonance imaging (mpMRI) and screening based on prostate-specific-antigen (PSA) testing has significantly improved the detection and localization of clinically significant PCa (Gleason ≥ 7, Prostate Imaging Reporting and Data System (PI-RADS) ≥ 3) [9, 13, 14]. The implementation of the PI-RADS has further enhanced diagnostic accuracy [15]. Patients with T3b PCa, characterized by seminal vesicle invasion (SVI), have considerably poorer PSA progression-free survival rates and overall outcomes than those with extraprostatic extension of PCa without SVI (T3a). The ability to infiltrate the SVs indicates a more aggressive tumor type [13, 14]. Therefore, accurate detection of SVI has significant prognostic value [16–18]. However, it remains difficult to distinguish between T3a and T3b PCa using biopsy, and mpMRI has a sensitivity of only 43.8% for the detection of SVI [13, 19]. Despite their clinical relevance, the SVs’ biophysical characteristics remain largely unexplored *in vivo*. Nevertheless, changes in their mechanical integrity may reflect underlying benign or malignant processes.

Magnetic resonance elastography (MRE) is a noninvasive imaging technique that allows *in vivo* quantification of tissue stiffness by externally inducing mechanical shear waves and visualizing their propagation through phase-contrast MRI. Applying a mathematical inversion algorithm allows for the transformation of shear wave-driven tissue displacement into quantitative stiffness maps. However, biological tissues often exhibit both solid-like and fluid-like behavior. This can be assessed *via* the frequency-dependent variation in shear wave speed. Furthermore, this enables the extraction of the phase angle φ, which reflects the relative contributions of solid-like and fluid-like behavior in response to shear wave propagation. MRE has shown significant clinical benefit in various organs, including the liver, kidney, breast, brain, intervertebral discs, and prostate [20–30].

Yet, to the best of our knowledge, MRE has not yet been systematically applied to the SVs. For diffusion-weighted imaging (DWI), Studies like those by Ren et al have demonstrated that areas of SVI exhibit significantly lower apparent diffusion coefficient (ADC) values compared with healthy SV tissue, and that combining T2-weighted imaging with DWI improves the detection of SVI [31, 32]. DWI is a standard component of PCa imaging because hyperintensity on high-*b*-value images with low ADC values indicates true diffusion restriction due to increased cellular density, which is a well-established marker of malignancy. Higher ADC values are related to lower Gleason scores in PCa. Exploring this relationship for the SVs may yield novel insights into tissue architecture and pathology, particularly in the context of PCa progression. Including DWI in our study therefore allows us to evaluate whether similar diffusion-based alterations can be observed and to compare these findings with MRE parameters, which may be particularly relevant for further improving the assessment of SVI [27, 32–38].

In this exploratory secondary analysis of a prospectively acquired dataset, we aim to characterize the viscoelastic properties of the SVs using multifrequency MRE and DWI. We analyzed three different clinical cohorts comprising healthy volunteers, men with benign prostatic hyperplasia (BPH), and patients with biopsy-confirmed PCa. We assessed shear wave speed (SWS) as a surrogate marker of tissue stiffness and the phase angle φ as an indicator of tissue fluidity in order to compare these parameters with ADC values derived from DWI, which have been normalized to the internal obturator muscle (nADC) [6, 26, 27, 29, 39–41].

By investigating biophysical properties within the SVs across health and disease states, this study seeks to investigate: (1) the feasibility of MRE in these small structures; and (2) to explore the relationship of SV biomechanical properties to BPH and PCa.

## Methods

### Study design and participants

This single-center secondary analysis of prospectively collected data was approved by the local ethical review board. Written informed consent was obtained from all participants following detailed information about the study. Our analysis of the SVs in the present study was performed to supplement already published data on MRE-based staging of BPH and PCa [6]. The data primarily stem from a previously published study on multiparametric MRI (mpMRI) and MRE in the prostate, in which the SVs were also delineable. While reference [6] focused on the viscoelastic properties of the prostate itself, the SVs were not analyzed.

To the best of our knowledge, the biomechanical characteristics of the SVs have not been systematically explored *in vivo*. Furthermore, the previous publication [6] did not include a healthy control group. In contrast, our study was performed to include healthy volunteers below the age of 40 years without known PCa or BPH. The current study therefore builds upon the work of Asbach et al [6] by performing a dedicated secondary analysis of the SVs using the same prospectively acquired imaging dataset, while supplementing it with newly acquired data from healthy subjects. By comparing SWS, φ, ADC, and nADC across these cohorts, we aim to characterize disease-related changes in SV biomechanics, particularly focusing on the potential relevance for PCa staging through the assessment of SVI.

Between May 2018 and March 2025, a total of 64 male subjects were included and subdivided into three groups: 25 patients with BPH, 14 with biopsy-proven PCa, and 25 healthy volunteers without known urogenital disease. In the PCa group, men were referred for MRI of the prostate either for surveillance of known PCa (active surveillance), for suspected PCa due to an elevated PSA level (> 4 ng/mL), or because of suspicious findings on transrectal ultrasound or during digital rectal examination. The decision to perform a prostate biopsy was made by the referring urologist based on clinical findings and PI-RADS score (see below), independent of MRE study participation [6].

**Fig. 1:**
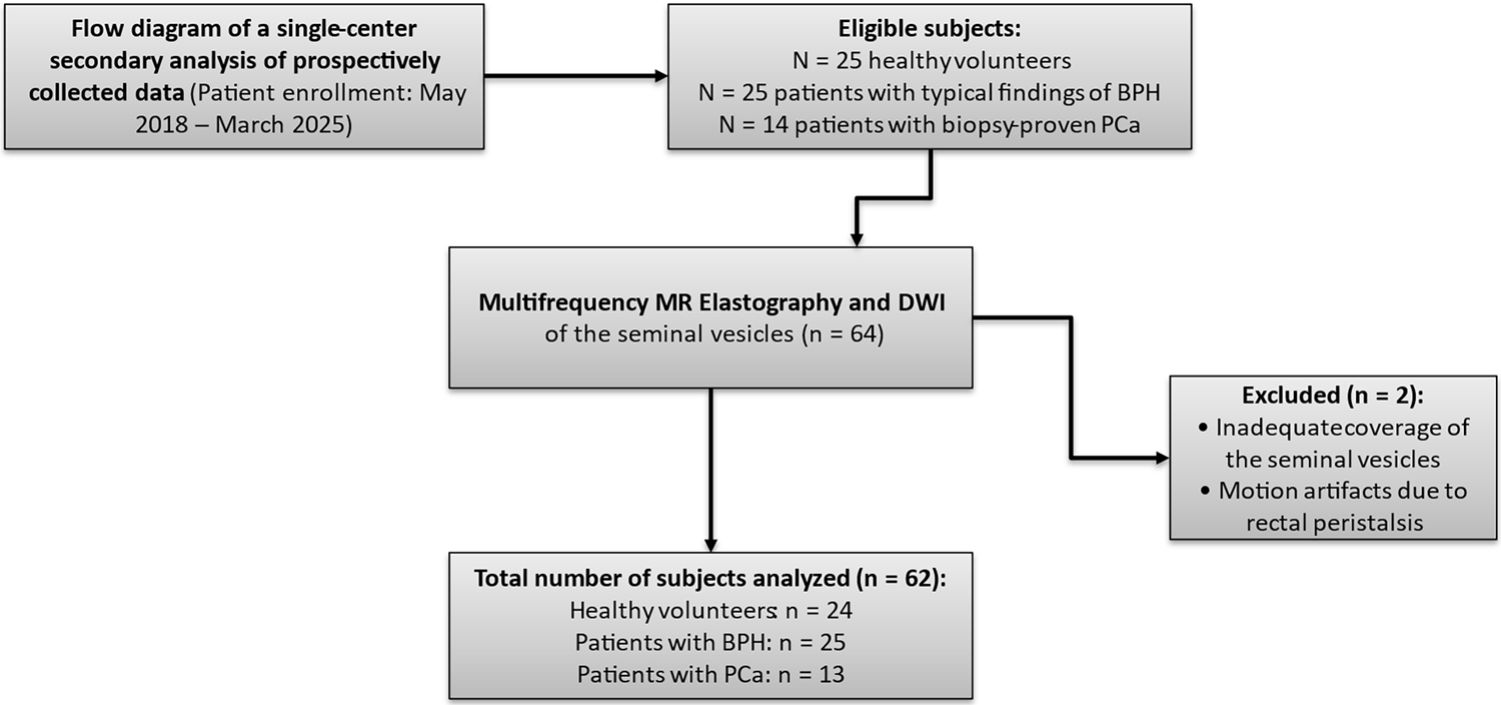
Flowchart of patient enrollment. BPH, Benign prostatic hyperplasia; PCa, Prostate cancer

Although the mpMRI scans in the PCa and BPH groups were originally acquired for clinical prostate assessment, they were secondarily repurposed for the current study to analyze the SVs. Consequently, two patients had to be excluded because the SVs were either not adequately covered in the imaging field or were affected by motion artifacts, most likely caused by rectal peristalsis. As a result, not all sequences—specifically the SWS-maps, ADC maps, and φ-maps—could be successfully segmented in every patient. Therefore, the final cohort consisted of 62 patients with varying availability of MRI sequences. To ensure a clear distinction between BPH and PCa, we did not include patients with unclear lesions in the current analysis (Fig. 1).

### MRI and MRE acquisition

All participants underwent mpMRI of the prostate according to PI-RADS guidelines (Version 2.0, 2015), which included T2-weighted imaging in axial and coronal planes and DWI in the axial plane. To ensure adequate SV filling, all participants were asked to abstain from sexual activity for at least 3 days prior to the MRI and MRE examinations.

DWI sequences were acquired with *b*-values of 0, 50, 500, and 1,000 s/mm², and a synthetic *b*-value of 1400 s/mm² was calculated. ADC maps were automatically generated by the MRI system using monoexponential fitting of all acquired *b*-values.

MRE was performed using external pneumatic drivers to generate vibrations of 60, 70, and 80 Hz in the body with a total image acquisition time of 4:19 min:s. The air-driven actuators were integrated into an anterior–posterior Velcro belt system, which was placed by a radiologist. A small cushion filled with sand was placed above the anterior driver to optimize wave introduction into the pelvis. During the prolonged study period, a minor adjustment in the actuator configuration was introduced. While the initial setup (as seen in reference [6]) utilized one anterior and two posterior pneumatic actuators, this was later extended to include a second anterior actuator for the healthy cohort, resulting in a total of four actuators. This modification followed recently published technical recommendations aimed at improving shear wave homogeneity in multifrequency MRE, particularly in deep-seated abdominal organs such as the pancreas [42]. This change was solely introduced to enhance shear wave coverage and overall image quality.

No systematic differences were expected in MRE-derived parameters (SWS and φ) between participants scanned with the original *versus* the updated actuator arrangement. The drive frequencies were chosen because they are known to yield stable results, while 50 Hz has demonstrated inferior performance [6, 42–45]. MRI was performed on a 3-T system (Magnetom Skyra; Siemens Healthineers) with an 18-channel phased-array surface coil positioned on the anterior pelvic wall and the spine array coil built into the patient table. Mechanical parameters derived from MRE, including SWS as a measure of stiffness and phase angle φ as a measure of fluidity [6, 39, 45], were obtained using a 3D motion-encoding scheme in axial orientation.

### MRE postprocessing

Tomoelastographic postprocessing of the multifrequency MRE data was conducted to generate quantitative viscoelastic maps. SWS, representing tissue stiffness, was calculated using the k-MDEV (wave number-based multifrequency dual elastovisco inversion) method. We chose to use k-MDEV for SWS mapping because of its robustness to noise [46]. However, k-MDEV does not account for wave attenuation or shadowing effects and is therefore not ideal for quantifying viscosity-related parameters such as φ [6, 46]. In contrast, MDEV incorporates wave attenuation and is thus more accurate in describing tissue fluidity [6, 39]. The k-MDEV and MDEV processing pipelines can be publicly accessed at bioqic-apps.charite.de [6]. SWS is commonly used as a surrogate marker of tissue stiffness because it reflects the square root of the storage modulus, a parameter frequently interpreted as mechanical stiffness in soft tissue [6, 29, 39–41]. Phase angle φ (phi), representing tissue fluidity, was calculated *via* MDEV inversion using in-house MATLAB scripts. φ is the ratio between the storage modulus (G′) and the loss modulus (G″), defined as φ = arctan(G″ / G′). The storage modulus represents the elastic, energy-storing properties of tissue, while the loss modulus reflects the viscous, energy-dissipating behavior. They define the complex shear modulus, which describes tissue stiffness and fluidity in MRE. A value of φ = 0, where G″ = 0, indicates purely elastic behavior, whereas φ = π/2, where G′ = 0, corresponds to purely viscous (fluid-like) behavior. φ ranges continuously from 0 (solid-like properties) to π/2 (fluid-like properties), and is therefore interpreted as a measure of fluidity [6, 39, 47, 48].

### Image analysis

Manual segmentation of the seminal vesicles was performed using ITK-SNAP (version 4.2.2; [49]; Penn Image Computing and Science Laboratory (PICSL), University of Pennsylvania) in all subjects by aligning the maps for SWS, φ, and ADC with anatomical T2-weighted images, magnitude images, and DWI [49]. Both SVs were defined as regions of interest (ROIs) for all three parameters. Quantitative values were automatically extracted from the segmented SVs using custom MATLAB (version R2024b) routines. For each parameter, the mean value of both SVs was calculated and used for intergroup comparisons. ADC values were normalized to the internal obturator muscle (nADC = ADC of the SVs / ADC of the muscle). To reduce potential bias, all segmentations were carried out by the same trained clinical investigator.

### Assessment of reproducibility and statistical analysis

Intraindividual reproducibility of MRI and MRE examinations of the SVs was assessed in five healthy volunteers who underwent imaging under identical conditions. After the first scan, each subject left the scanner room and was repositioned before undergoing a second scan. Intraclass correlation coefficients (ICCs) were calculated to quantify agreement between results obtained in the left and right SV, as well as between the two repeated imaging sessions. Additionally, Bland–Altman analyses were performed to visualize the mean difference (bias) and the 95% limits of agreement between paired measurements, offering a graphical assessment of measurement consistency.

All statistical analyses were performed using RStudio (Version 4.5.0). ChatGPT (OpenAI) was used as a non-decision-making AI support tool. Code syntax for descriptive and inferential statistics was refined with the assistance of AI to ensure reproducibility and improve data visualization. Statistical significance was defined as *p* < 0.05. Mean values were calculated for the continuous variables SWS, φ, and ADC in patients with PCa, patients with BPH, and healthy controls, and are presented as mean ± standard deviation. To assess differences between the three subject groups (healthy, BPH, and PCa) for each parameter (SWS, φ, and ADC), we performed Kruskal–Wallis tests, followed by Dunn’s *post hoc* tests (with Holm correction). Based on these results, we generated boxplots including jittered data points for graphical representation. The Shapiro–Wilk test was used to assess the normality of the data.

Pearson correlation coefficients were computed to evaluate relationships between biophysical imaging parameters (SWS, φ, ADC and nADC) and their associations with age and body mass index (BMI). To assess potential differences between PCa patients with and without SVI, Welch *t*-test was performed to compare these two subgroups.

## Results

The total study population analyzed included 62 subjects (mean age: 53.3 years; range: 23–88 years) subdivided into 13 with biopsy-proven PCa, 25 with BPH, and 24 healthy volunteers. Participants were included in the analyses if at least one of the two bilateral ROI measurements (right or left) was valid. When both of the bilateral ROI measurements were of sufficient quality, we used the mean of the left and the right SV. The Shapiro–Wilk test indicated that SWS, φ, and ADC were normally distributed in the subgroups of healthy subjects and patients with BPH. For patients with PCa, φ and ADC values were normally distributed. However, SWS values significantly deviated from normality. In order to reduce bias, we performed Kruskal–Wallis and Dunn tests for all groups to account for the deviation from normality. We conducted these analyses to investigate group differences in SWS, φ, ADC and nADC among healthy volunteers, patients with BPH, and patients with PCa.

### Kruskal–Wallis and Dunn analyses

ADC values did not differ significantly among the three groups. Although a tendency toward higher values was observed in PCa patients compared to healthy controls, this difference did not reach statistical significance (*p* = 0.089). A similar trend was found for SWS values (*p* = 0.201).

nADC values were significantly lower in both BPH and PCa patients compared to healthy controls. The decrease was statistically significant for the comparison between healthy subjects and the PCa group, as well as the comparison between healthy controls and BPH patients (*p* < 0.001 for both). However, the difference between the BPH and PCa groups was not significant (*p* = 0.233).

For φ, significantly higher values were observed in subjects with BPH and PCa compared to healthy controls (*p* = 0.022 and *p* = 0.019, respectively), suggesting increased viscous properties in pathologically altered SVs. No significant difference was found between patients with BPH and PCa (*p* = 0.499).

**Fig. 2:**
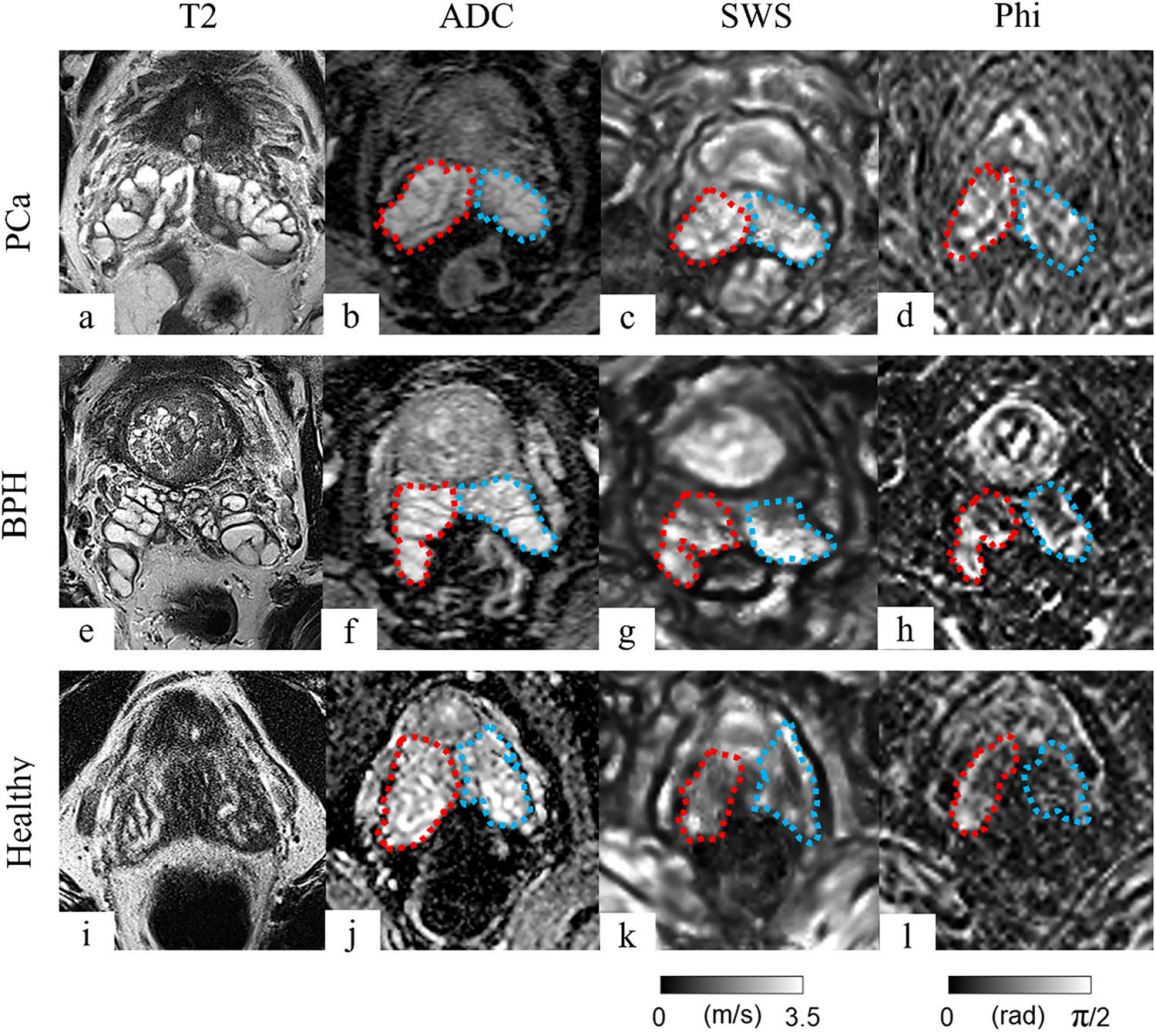
Representative multiparametric MRI and MR elastography sequences. The first row shows images from a patient with prostate cancer (PCa) (**a**–**d**), the second row from a patient with benign prostatic hyperplasia (BPH) (**e**–**h**), and the third row from a healthy volunteer (**i**–**l**). The right seminal vesicle is encircled in red, and the left seminal vesicle in blue

Representative mpMRI and MRE images from patients with PCa and BPH and from a healthy subject are shown in Fig. 2.

In summary, φ appears to discriminate pathological alterations from healthy SV tissue, while both SWS and ADC only showed a non-significant trend toward higher values in PCa. Descriptive statistics and the results of the Kruskal–Wallis test with Dunn’s *post hoc* comparisons are presented in Table 1.

**Table 1:** Descriptive statistics of seminal vesicle parameters

Group	ADC Mean ± SD	ADC Median [IQR]	nADC Mean ± SD	nADC Median [IQR]	φ Mean ± SD	φ Median [IQR]	SWS Mean ± SD	SWS Median [IQR]
Healthy	1430.7 ± 287.9	1351.9 [490.2]	3.75 ± 1.12	3.43 [1.05]	0.748 ± 0.108	0.738 [0.140]	1.990 ± 0.264	2.043 [0.377]
BPH	1529.1 ± 93.2	1496.9 [134.1]	2.68 ± 0.67	2.75 [1.08]	0.822 ± 0.076	0.803 [0.100]	2.051 ± 0.229	2.031 [0.303]
PCa	1604.7 ± 172.3	1628.4 [251.5]	2.40 ± 0.45	2.47 [0.40]	0.837 ± 0.062	0.849 [0.076]	2.251 ± 0.404	2.139 [0.353]

Values are presented as mean ± SD or median [IQR]

*ADC* Apparent diffusion coefficient, *BPH* Benign prostatic hyperplasia, *nADC* Normalized ADC, *PCa* Prostate cancer, *SWS* Shear wave speed, *φ* Loss angle

### Test-retest reproducibility

**Fig. 3:**
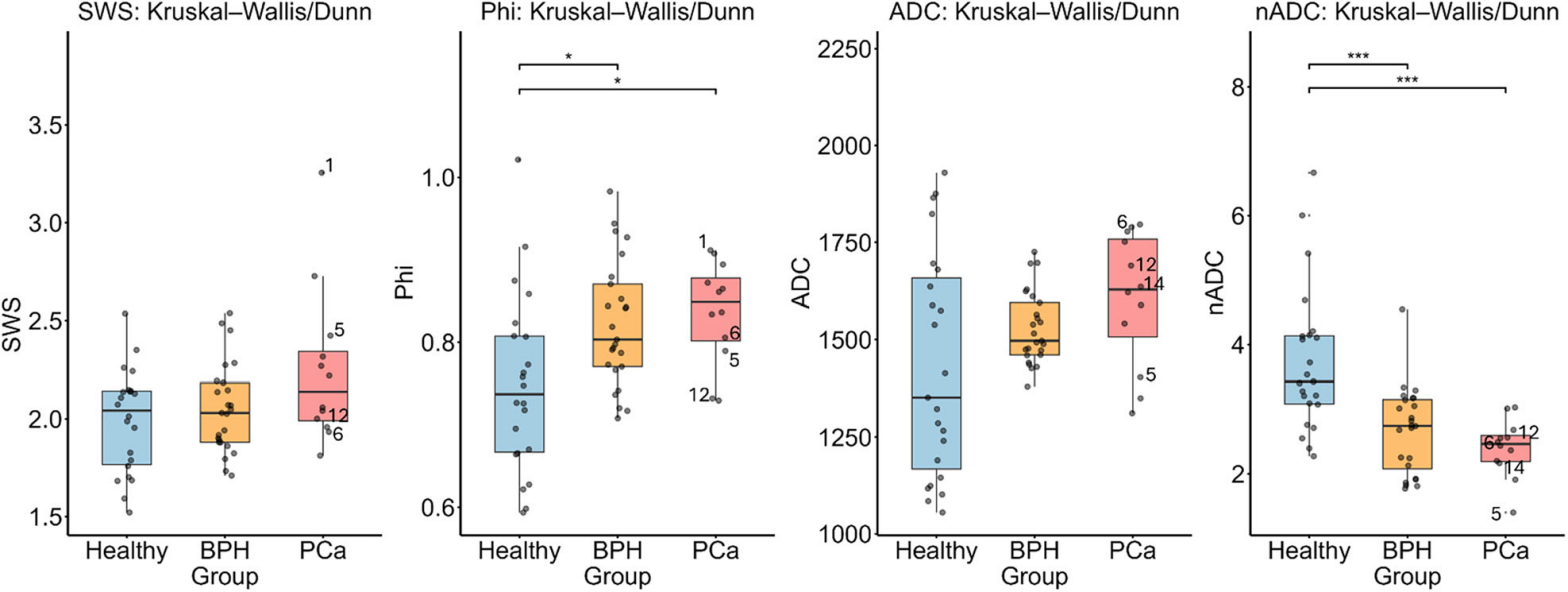
Boxplots of MRE (SWS, m/s; φ, rad) and DWI (ADC, ×10^−3^ mm^2^/s; nADC, unitless) parameters in patients with prostate cancer (PCa), patients with benign prostatic hyperplasia (BPH) and healthy controls. PCa patients with SVI were labeled by their patient ID (1, 5, 6, 12 and 14). Brackets represent statistically significant differences (*p* < 0.05) Statistical significance is denoted as follows: * *p* < 0.05, ** *p* < 0.01, *** *p* < 0.001 [6]

**Fig. 4:**
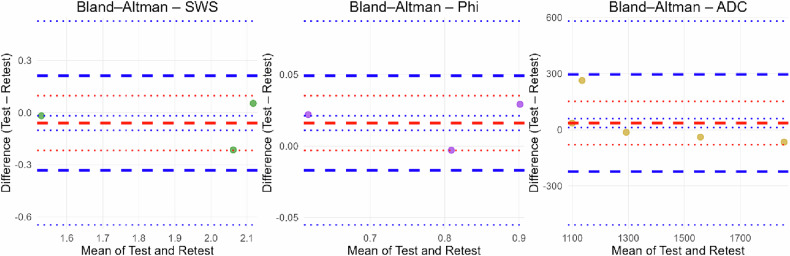
Bland–Altman plots illustrating test-retest reproducibility for SWS, φ, and ADC. The bold red dashed line marks the mean difference (bias). The bold blue dashed lines denote the 95% limits of agreement. The thin dashed lines (red and blue) delineate the 95% confidence intervals for the mean difference and the limits of agreement [54]

**Fig. 5:**
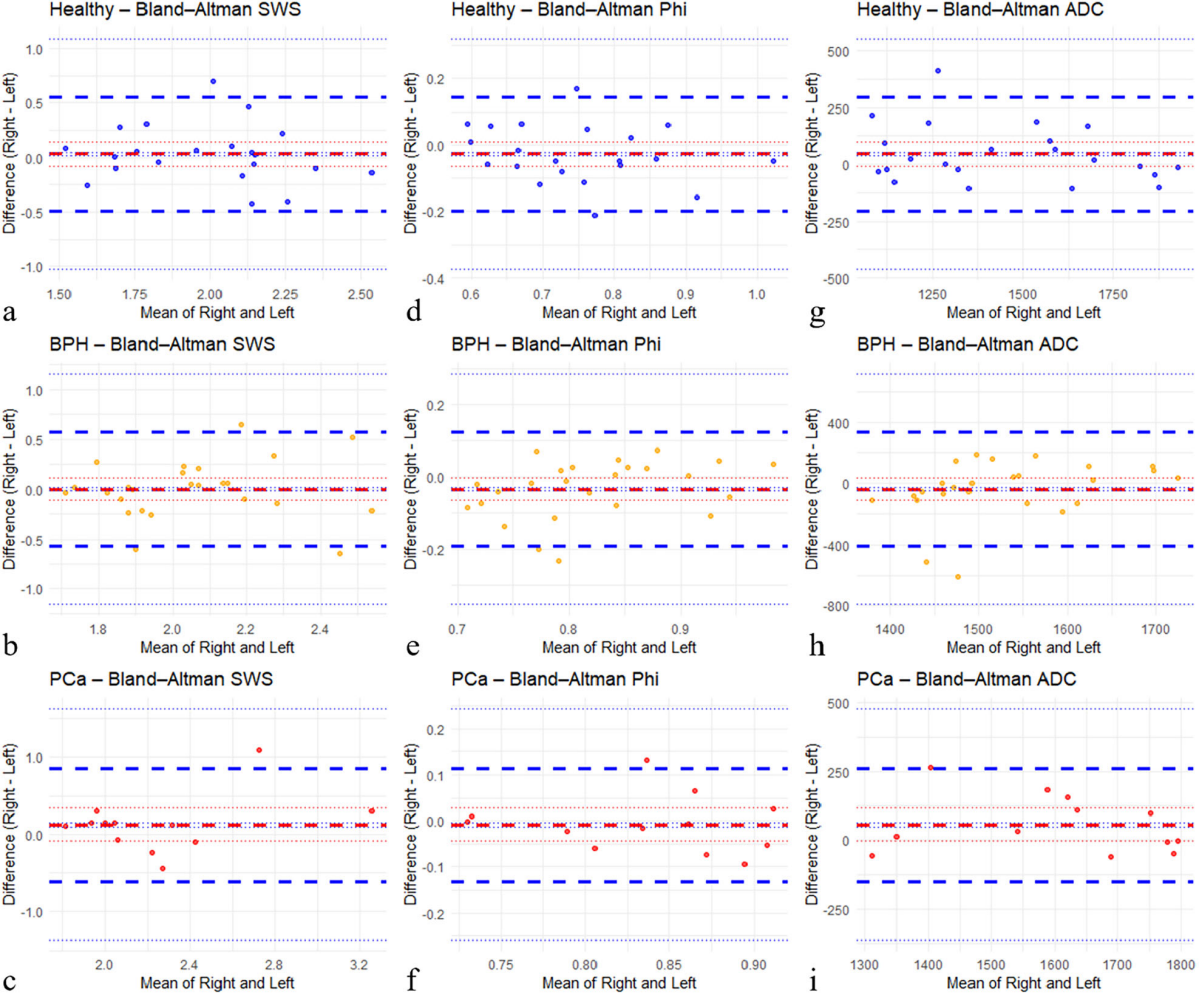
Bland–Altman plots illustrating bilateral agreement for SWS (**a**–**c**), φ (**d**–**f**), and ADC (**g**–**i**) across groups. The bold red dashed line marks the mean difference (bias). The bold blue dashed lines denote the 95% limits of agreement. The thin dashed lines (red and blue) delineate the 95% confidence intervals for the mean difference and the limits of agreement [54]. ADC, Apparent diffusion coefficient; SWS, Shear wave speed; φ, Phase angle

Test-retest consistency was evaluated by examining five healthy controls a second time and calculating ICCs. ADC values from all five subjects were included in the analysis. For SWS and φ values, both the original and retest images of two volunteers could not be incorporated due to motion artifacts, most likely caused by rectal peristalsis. For both ADC and SWS, the ICCs (0.926 and 0.925) indicated strong agreement between repeated measurements. The highest reproducibility was observed for φ with an ICC of 0.989. Overall, consistently high ICC values across all parameters indicated excellent reproducibility. Additionally, paired *t*-tests showed no statistically significant differences between test and retest values for all parameters (SWS: *p* = 0.915, φ: *p* = 0.993, ADC: *p* = 0.952), further supporting the stability and reproducibility of the measurements (Figs. 3–5).

### Bilateral reliability

Bilateral agreement of SV measurements was evaluated using ICCs, Pearson’s correlations, and mean absolute differences (MADs). Healthy subjects showed near excellent left–right agreement for ADC (ICC = 0.896, *r* = 0.908, MAD = 94.7), whereas bilateral consistency was only moderate for SWS (ICC = 0.619, *r* = 0.616, MAD = 0.19) and φ (ICC = 0.721, *r* = 0.740, MAD = 0.07). Left–right agreement was considerably lower in patients with BPH, especially for ADC (poor reproducibility, ICC = -0.018, *r* = -0.019, MAD = 128.3). SWS (ICC = 0.433, *r* = 0.424, MAD = 0.21) and φ (ICC = 0.533, *r* = 0.589, MAD = 0.06) showed poor to moderate agreement. For patients with PCa, SWS (ICC = 0.642, *r* = 0.673, MAD = 0.27) and φ (ICC = 0.608, *r* = 0.596, MAD = 0.05) also demonstrated moderate bilateral agreement, whereas ADC (ICC = 0.802, *r* = 0.837, MAD = 85.8) revealed good reliability.

In summary, ADC values showed the highest bilateral consistency for healthy subjects and patients with PCa. SWS and φ revealed only moderate but stable reproducibility across all groups. Bilateral agreement was lowest in patients with BPH, particularly for ADC, suggesting that pathological alterations of the prostate and SVs may compromise left–right measurement reproducibility.

### Comparison of PCa patients with *vs*. without SVI

Five of the patients in the PCa group had SVI confirmed by mpMRI, one of whom had to be excluded. Mean values did not differ significantly between the two subgroups for SWS (difference = 0.248, 95% CI: -1.157 to 0.661, *p* = 0.481), φ (difference = -0.039, 95% CI: -0.072 to 0.150, *p* = 0.400), or ADC (difference = 37.217, 95% CI: -286.340 to 211.906, *p* = 0.734). Although no statistically significant differences were observed between PCa patients with and without SVI in terms of SWS, φ, ADC and nADC, this result must be interpreted with caution. Due to the small sample sizes in both subgroups, the statistical power of this comparison is limited, and the analysis must be considered exploratory.

### Correlation analyses

Overall, no statistically significant age- or BMI-related effects in the mechanical properties of the SVs were observed across parameters and groups. Significant correlations between SWS and φ were observed in both healthy subjects (*p* = 0.006) and BPH patients (*p* < 0.001), whereas no other significant associations were found across the remaining parameters and cohorts.

## Discussion

To the best of our knowledge, this is the first study to characterize the mechanical and viscoelastic properties of the SVs, including tissue stiffness and fluidity, using MRE. We aimed to investigate quantitative measures such as SWS, φ, ADC and nADC across healthy individuals, patients with BPH, and patients with PCa. Among the assessed metrics, φ proved to be a sensitive marker of pathological alterations, with significantly higher values observed in both BPH and PCa compared to healthy controls.

This finding implies that prostatic pathologies, whether due to BPH or PCa, are associated with increased viscosity of the SVs. Multiple studies have previously highlighted the important role of high tumor fluidity in promoting infiltration of surrounding tissue [6, 45, 50, 51]. This may suggest that either higher fluidity of the SVs increases the risk for SVI or SVI increases tissue fluidity. However, the second hypothesis does not account for higher tissue fluidity in the BPH group. Further studies could provide valuable insights into which viscoelastic properties increase the risk for tumor infiltration.

Our results also showed significantly lower nADC values in BPH and PCa patients compared to healthy subjects. The reduction observed in the PCa group aligns with the commonly reported decrease in ADC in malignant tissue, with normalized ADC values being additionally calculated to reduce measurement variability inherent to conventional ADC estimation [52]. In our study, diffusion metrics were assessed within the SVs rather than within intraprostatic tumor tissue. In cases of SVI, dense tumor cells may replace secretory epithelial structures, reduce luminal fluid storage, and increase diffusion restriction through increased cellularity, thereby leading to lower nADC values in infiltrated SVs [32]. Lower nADC values in BPH may reflect chronic outflow obstruction and enhanced stromal components due to tissue remodeling, resulting in the reduction of luminal space [53]. For SWS, only non-significant trends toward higher levels in PCa were observed. In contrast to φ, SWS may therefore be less effective in discriminating pathological from normal tissue. The high consistency of all measurements, especially the excellent test-retest reliability of φ, highlights the methodological robustness and reproducibility of multifrequency MRE for examining the SVs.

The feasibility of MRE has also been established for other organs [6, 25, 54]. However, most MRE studies of the liver, kidney, prostate, and breast have mainly focused on tissue stiffness [20–23, 45]. In contrast, studies like those by Reiss-Zimmermann et al and Asbach et al support our findings that tissue fluidity may be even more important in certain organs, such as the brain, prostate, and SVs [6, 24, 45]. This is particularly relevant for the SVs, as certain conditions, such as amyloidosis, may cause wall thickening that may mimic cancer. Across several tumor entities, studies have demonstrated that malignant lesions generally show elevated φ compared to healthy tissue. Fluidization is associated with cellular unjamming and proliferation, as well as vascularization and necrosis. Streitberger et al interpret increased fluid-like behavior to indicate internal cellular rearrangement due to reduced mechanical resistance to deformation. Therefore, higher tissue fluidity could potentially be a predictive factor for aggressiveness, invasive potential, and metastatic spread in malignant diseases [45, 50, 51]. Other types of cancer, such as bladder or rectal cancer, can also infiltrate the SVs. Further studies analyzing the SVs in the context of these cancers may provide valuable information on a range of different disease entities [55].

Bilateral agreement varied among groups: we observed good left–right consistency for ADC in healthy controls and patients with PCa, but reproducibility was severely impaired in BPH. The latter could indicate heterogeneous structural remodeling of SVs caused by BPH, or asymmetric seminal fluid flow through the excretory duct due to structural changes in the prostate. Additional subgroup analyses showed no significant differences across all parameters between PCa patients with and without SVI. This may be because, according to the literature, mpMRI only has 43.8% sensitivity for detecting SVI [19]. Thus, some patients may have been misclassified as negative for SVI due to technical limitations. Additionally, the small sample sizes in both subgroups limit the statistical power of this comparison. Abnormal alterations of tissue fluidity may also occur in early stages of disease and may not solely be attributable to direct tumor infiltration, which is supported by the observation that φ is also significantly higher in patients with BPH compared to healthy controls. Further studies including more participants with PCa could provide more conclusive insights in the future. Across the three groups, no significant associations with BMI or age were observed. This finding may indicate a relative stability of SV viscoelastic properties across the lifespan, suggesting that MRE could have diagnostic value across different body types and age groups.

Our study has several limitations. First, we present a secondary analysis of prospectively collected data. The mpMRI and MRE sequences were originally acquired to directly analyze the prostate, resulting in the SVs not always being optimally covered. Consequently, the number of slices that could be segmented varied across patients, reducing comparability across groups. Additionally, ADC values were derived at a different slice orientation compared with SWS and φ, sometimes resulting in inadequate coverage and inconsistencies in slice angulation. Therefore, only one SV could be segmented in some instances, which impaired direct comparability. The original study included only PCa lesions > 10 mm in diameter and predominantly high-grade PCa patients, which may have introduced bias. Moreover, PCa could not be definitely ruled out in all patients with BPH, as those with likely BPH did not undergo systematic biopsy [6]. Furthermore, some participants showed motion artifacts, attributable to rectal peristalsis. Additional artifacts may have resulted from heterogeneous rectal filling and variability in anatomical positioning.

In conclusion, MRE of the seminal vesicles is technically feasible, reliable and a promising approach for characterizing the viscoelastic properties in pathologies affecting both the prostate and the SVs. Particularly, tissue fluidity seems to be a promising imaging marker for detecting SV abnormalities, potentially indicating microscopic changes in tissue organization and extracellular matrix composition. In summary, these results warrant further investigation in larger studies to validate and extend our findings.

## Data Availability

All datasets generated or analyzed in this study are available on request from the corresponding author.

## Abbreviations

ADC: Apparent diffusion coefficient
BPH: Benign prostatic hyperplasia
CI: Confidence interval
DWI: Diffusion-weighted imaging
G″: Loss modulus
G′: Storage modulus
ICC: Intraclass correlation coefficient
MAD: Mean absolute difference
MDEV: Multifrequency dual elastovisco inversion
mpMRI: Multiparametric magnetic resonance imaging
MRE: Magnetic resonance elastography
MRI: Magnetic resonance imaging
nADC: Normalized apparent diffusion coefficient
PCa: Prostate cancer
PI-RADS: Prostate Imaging Reporting and Data System
PSA: Prostate-specific antigen
ROI: Region of interest
SVI: Seminal vesicle invasion
SVs: Seminal vesicles
SWS: Shear wave speed
φ: Phase angle
