# Correction: Feasibility of multifrequency MR elastography of the seminal vesicles in healthy men, benign prostatic hyperplasia, and prostate cancer

**DOI:** 10.1186/s41747-026-00772-5

**Published:** 2026-07-09

**Authors:** Kevin Vatter Davis, Hans-Martin Thiess, Patrick Asbach, Eugenio Tiberi, Timm Denecke, Bernd Hamm, Bernhard Gebauer, Jens Vogel-Claussen, Ingolf Sack, Rolf Reiter

**Affiliations:** 1 Department of Radiology, Charité–Universitätsmedizin Berlin, Corporate Member of Freie Universität Berlin and Humboldt-Universität zu Berlin, Hindenburgdamm 30, Berlin, Germany; 2 Department of Urology, BG Unfallkrankenhaus Berlin, Berlin, Germany; 3 Department of Diagnostic and Interventional Radiology, University of Leipzig, Leipzig, Germany; 4 Berlin Institute of Health at Charité–Universitätsmedizin Berlin, BIH Biomedical Innovation Academy, BIH Charité Digital Clinician Scientist Program, Charitéplatz 1, Berlin, Germany

**Correction to: European Radiology Experimental**

10.1186/s41747-026-00713-2, published online 21 April 2026

Following publication of the original article, the figure caption of Figure 3 has been updated to include the measurement units of the displayed parameters (SWS, φ, ADC, and nADC). This correction does not affect the scientific content of the article.

The original article has been corrected.

